# Patients’ rights in physicians’ practice during Covid-19 pandemic: a cross-sectional study in Romania

**DOI:** 10.1186/s12910-023-00935-8

**Published:** 2023-07-26

**Authors:** Maria Cristina Plaiasu, Dragos Ovidiu Alexandru, Codrut Andrei Nanu

**Affiliations:** 1 Doctoral School, University of Medicine and Pharmacy of Craiova, 2 Petru Rares St, Craiova, 200349 Romania; 2 Department of Medical Informatics and Biostatistics, University of Medicine and Pharmacy of Craiova, 2 Petru Rares St, Craiova, 200349 Romania; 3 Department no. 14 of Orthopedics, Anesthesia and Intensive Care, University of Medicine and Pharmacy “Carol Davila”, 37 Dionisie Lupu St., Sector 2, Bucharest, 020021 Romania

**Keywords:** Physicians’ practice, Patients’ rights, Legal compliance, covid-19

## Abstract

**Background:**

Although the Covid-19 epidemic challenged existing medical care norms and practices, it was no excuse for unlawful conduct. On the contrary, legal compliance proved essential in fighting the pandemic. Within the European legal framework for the pandemic, patients were still entitled to be treated equally, by a specialized physician, with the possibility of seeking a second medical opinion, in a confidential setting, following prior and informed consent. This study examines physicians’ practices regarding patients’ rights during the Covid-19 pandemic and the effects of age, experience, and specialty on physicians’ behavior and preferences. Additionally, it explores the nexus of malpractice complaints, malpractice fear, and legal compliance.

**Methods:**

A cross-sectional study was conducted on a convenience sample of attending physicians and general practitioners to assess compliance with patients’ rights regulations. Respondents were physicians practicing in private and public settings in Southwestern Romania from July 2021 to May 2022.

**Results:**

396 attending physicians and 109 general practitioners participated in the research. Attending physicians acknowledged patients’ rights in 55.7% of statements, while general practitioners showed a slightly higher level of compliance at 59.9%. Emergency and Anesthesia and Intensive Care physicians showed the lowest compliance. There were no significant behavioral differences based on physicians’ age, years in practice, work sector, or location. However, when faced with the question of prioritizing treatment for patients with similar medical conditions, 46.2% of attending physicians reported favoring the younger patients. This preference was common among physicians under 39. Additionally, over half of the attending physicians reported working outside their area of expertise due to staff shortages. Malpractice fear was high among physicians, although unrelated to patients’ claims, legal compliance, or working outside the scope of practice. It resulted in pressure and behavioral changes.

**Conclusion:**

Adherence to patients’ rights was low during the Covid-19 pandemic. Physicians could benefit from educational and administrative support to ensure better legal compliance. Further research is needed to determine if this behavior persists beyond the pandemic context.

**Supplementary Information:**

The online version contains supplementary material available at 10.1186/s12910-023-00935-8.

## Introduction

Although the Covid-19 epidemic was an unusual event that challenged existing medical care norms and practices, often beyond practitioners’ control, it was no excuse for unlawful conduct. On the contrary, legal compliance proved essential in fighting the pandemic [1]. Restriction of certain liberties was decided in order to protect public health [2–4]. In the medical field, patients’ consumer rights to choose and access non-critical healthcare services and elective surgery were limited in certain circumstances [5–8]. The rules on sharing patients’ confidential data were relaxed during the pandemic to help fight it. As a result, healthcare organizations were authorized to share patient information without prior consent. In England, the National Health Service (NHS), for instance, was granted the authority to utilize patient records to identify and prioritize highly vulnerable individuals for early vaccination and shielding [9, 10]. However, patients’ basic rights were not fundamentally altered [11, 12]. Although European countries’ legislation imposed sanctions for failure to isolate or refusal to provide bodily samples, it did not permit forced medical interventions. Within the European legal framework for the pandemic, patients were still entitled to be treated equally, by a specialized physician, with the possibility of seeking a second medical opinion, in a confidential setting, following prior and informed consent [13].

Achieving legal compliance during a crisis can be as difficult as it is important. Physicians experienced professional challenges [14], stress [15], depersonalization, and emotional exhaustion [16–18], which affected the quality of care provided to patients [19]. Additionally, factors such as trust, emotions, threat and risk perceptions influenced physicians’ compliance with Covid-19 guidelines and legal requirements [20]. Studies showed a decline in the quality of care for patients with chronic diseases [21, 22], elderly and incapacitated patients [23]. The shared decision-making process was affected [24], and patients’ satisfaction decreased [25, 26]. Additionally, the pandemic raised concerns about paternalism resurgence and discriminatory practices related to resource allocation [27, 28].

In Europe, there are significant variations in awareness and implementation of patients’ rights among European nations, despite the existence of a uniform framework established by European conventions and directives pertaining to healthcare [13]. The Romanian law on patient rights focuses primarily on traditional patient rights, as stated in the Oviedo Convention. The shared decision-making process and the advanced directives are not regulated. In practice, most physician-patient relationships are dominated by paternalistic influences, and enforcement of patients’ rights remains weak [1]. Studies conducted before the Covid-19 pandemic showed inadequate physicians’ compliance with patients’ rights [20, 29].

## Methods

This study aims to contribute to new knowledge regarding physicians’ practices in a crisis, focusing on their adherence to patients’ rights during the Covid-19 pandemic. This research examines the effects of age, experience, and specialty on physicians’ legal compliance and treatment preferences for patients in the event of resource scarcity. Additionally, it explores the nexus of malpractice complaints, malpractice fear, and legal compliance. Low levels of legal compliance were anticipated due to the pandemic conditions and the absence of a medical law university curriculum. Moreover, high pressure on Anesthesia and Intensive Care and emergency physicians [30] was expected to cause lower legal compliance and increased fear of malpractice.

### Settings and study design

We conducted a cross-sectional study among Romanian attending physicians and general practitioners during the Covid-19 pandemic. The study was carried out from July 2021 to May 2022 in Southwestern Romania, covering the contiguous counties of Dolj and Olt. The region is served by sixteen public hospitals, providing medical services to approximately one million residents. Dolj county is economically more advanced than Olt county, with twice as many businesses and a larger population [31].

For the context, the country experienced its first peak in November 2020, with over 8000 daily confirmed cases, followed by a second peak in October 2021, with almost 15,000 daily confirmed cases. A third peak followed this in February 2022 with over 30,000 daily confirmed cases [32]. Romania demonstrated a highly inefficient health system and allocated insufficient resources, especially during the second wave of the pandemic [33]. In 2020, Romania had one of the lowest ratios of practicing physicians to population (330 per 100,000 people) and one of the highest proportions of younger physicians under 35 years old among European countries [34]. In 2021, Anaesth Intensive Care and emergency physicians accounted for 8% of all Romanian attending physicians [35]. The Romanian medical system highly depends on public funding [36], and the health network is predominantly built in urban areas. Most attending physicians work in public hospitals or divide their time between public and private hospitals, whereas most general practitioners work in private practices.

The study uses a previously validated questionnaire to assess physicians’ legal compliance with patients’ rights regarding informed consent, confidentiality, access to medical data, second opinion, non-discrimination, and being cared for by a competent physician [29]. The survey was developed through a literature review and first tested on Romanian physicians, then on medical students, before the current study’s refinement [37]. The questionnaire was based on the provisions of Law No. 46/2003 on Patients’ Rights, which transposes the Oviedo Convention into Romanian law. The questionnaire consisted of three sections, with 18 questions for attending physicians (Appendix A) and 14 for general practitioners (Appendix B). The first section collected data regarding age, experience, specialty, work sector, geographic region, and place of employment. The second section included multiple-choice questions regarding physicians’ behavior toward patients. The final section gathered information on work conditions, patients’ claims, and malpractice risks. The questionnaire was anonymous. Physicians were instructed to choose answers that best reflected their current routine practices. They were provided with the option to choose multiple answers or none at all.

### Participants

The research targeted attending physicians who were providing patient care in public and private hospitals and general practitioners who were practicing in private practices during the second and third peaks of the pandemic. The study excluded pathologists, laboratory physicians, and researchers due to their limited direct engagement with patients in their routine practice. Psychiatrists were excluded based on their adherence to specific legislation, particularly regarding informed consent. Furthermore, residents were not included in the study as they operate as trainees under the guidance and supervision of senior physicians, resulting in limited decision-making autonomy. Attending physicians were classified into broad medical fields: surgical specialties, non-surgical specialties, obstetrics and gynecology, emergency medicine, and anesthesia and intensive care. The classification was based on legal criteria and specialty specifics. The study used a convenience sample. To encourage participation and eliminate sample biases, we designed an introduction section to the questionnaire to explain the objective, kept it to a minimum of questions, and made it simple for attending physicians to collect and return them [38].

We used an online questionnaire for general practitioners and a printed form for attending physicians. The Association of General Practitioners from Dolj county helped distribute it among its 300 members. The sample size was set at 169 participants, with a 95% confidence interval, 5% margins of error, and 50% population proportion. The questionnaire was available online from July to October 2021.

According to the College of Physicians, in 2021, there were 2830 physicians registered in Olt and Dolj counties. Therefore, we calculated a sample size of 339 participants using a confidence interval of 95%, 5% margins of error, and 50% population proportion. We approached public and private hospitals to participate in the study. Eight of the largest public hospitals in Dolj and Olt’s counties and two private hospitals in Dolj’s accepted to participate. Due to pandemic conditions, our access to hospital wards was limited. Therefore we sought support from ward administrators. Between November 2021 and May 2022, 563 printed questionnaire forms were provided to head wards, placed in on-call rooms, or handed directly to physicians.

### Data analysis

We compared physicians’ answers to the legal requirements to determine compliance with patient rights. We assigned one point for each accurate response and calculated a score for each physician based on their responses. For the question regarding discriminatory practices, we considered the absence of any selection as the correct response. Questionnaires with missing demographic information or responses to some or all self-evaluation questions wereconsidered valid. Additionally, questionnaires only comprising demographic or self-evaluation checks were deemed invalid. We limited our analysis to questions related to patients’ rights and excluded question 12, which did not pertain to patient rights, and question 15, which was already addressed in a previous paper (Appendix A) [39]. Regarding the internal consistency of the questionnaire, it was designed as a multidimensional scale to evaluate a broad perspective on physicians’ practice. Therefore a high Kuder-Richardson Reliability Coefficient was not the primary objective. Instead, we calculated mean inter-item correlations for the related items as recommended by Briggs and Cheek (1986) [40], and these scores were no less than 0.2.

We used Microsoft Excel (Microsoft Corp., Redmond, WA, USA), the XLSTAT add-on for Microsoft Excel (Addinsoft SARL, Paris, France), and IBM SPSS Statistics 29.0.0.0 to process the data (IBM Corporation, Armonk, NY, USA). Normality tests and complex statistical tests (Chi-Square, Kruskal-Wallis, Friedman) were performed using SPSS. The Shapiro-Wilks test was used to assess the data’s normality. The nonparametric Kruskal-Wallis test for multiple pairwise comparisons with Bonferroni correction was used because the study included numerical comparisons between more than two groups that did not have a standard (Gaussian) distribution. The Chi-square test was used to assess categorical data to identify a link (association or influence) between two parameters generated by the cross-tabulation of two categorical variables collected. ChatGPT and Grammarly were used exclusively for grammar and English language editing.

## Results

The questionnaires were filled out by 506 physicians, including 396 attending physicians and 110 general practitioners. The response rate exceeded the sample size for attending physicians and was 65% for general physicians. We excluded one online questionnaire for the general practitioners for lack of consent and improper completion.

Attending physicians’ average age was 43.3 ± 10.6 years, and their average years in practice was 16.1 ± 10.6. The average age of general practitioners was 56.2 ± 7.6 years, and their professional experience was 29 ± 8.7 years. 79.8% of general practitioners worked in urban, while 20.2% worked in rural areas. The baseline characteristics of the respondents are shown in Table 1.

**Table 1:** Baseline characteristics of the physicians

Variables	Attending Physicians		General Practitioners	
	No	%	No	%
Specialty				
Anesth. Intensive Care	24	6.1	N/A	
Surgical	96	24.2	N/A	
Non – surgical	181	45.7	N/A	
ObGyn	71	17.9	N/A	
Emergency	24	6.1	N/A	
Age (years)				
< 30	27	6.8	0	0
30–39	129	32.6	2	1.8
40–49	102	25.8	21	19.3
50–59	74	18.7	43	39.4
> 60	31	7.8	43	39.4
N/A	33	8.3	0	0
Years in practice				
1–5 6–10 11–15 16–20 21–25 26–30 > 30 N/A	75	18.9	1	0.9
	60	15.2	2	1.8
	58	14.6	6	5.5
	48	12.1	10	9.2
	52	13.1	18	16.5
	37	9.3	20	18.3
	31	7.8	50	45.9
	35	8.8	2	1.8
Work sector				
Public Private Both	208	52.5	N/A	
	15	3.8	N/A	
	173	43.7	N/A	
Geographic area				
Urban Rural	388	98	87	79.8
	8	2	22	20.2
Location				
Dolj Olt	305	77	109	100
	91	23	0	0

Due to the small number of rural physicians, we found it inconclusive to evaluate attending physicians according to the geographic area criteria. Likewise, we categorized attending physicians into two groups: physicians working exclusively in the public sector and physicians working in the private sector, either exclusively in the private sector or in both private and public sectors.

### Physicians’ compliance with patients’ rights

Attending physicians acknowledged patients’ rights in 55.7% of statements, while general practitioners showed a slightly higher level of compliance in 59.9%. The analysis of physicians’ responses to each presented statement showed that some patients’ healthcare rights were respected more than others (Table 2).

**Table 2:** Physicians’ Compliance with Patients’ Rights

Component of Patient’s Right	Attending Physicians No (%)		General Practitioners No (%)	
Not to be discriminated against due to age, nationality, or income	195	49.2	54	49.5
To be treated by a physician with legal and medical competency	109	27.5	59	54.1
To refuse treatment	105	26.5	62	56.9
To receive a second medical opinion	288	72.7	N/A	
To be informed about the medical act	389	98.2	107	98.2
To provide explicit (written) consent for the medical act				
Consent for biological samples	225	56.8	61	56
Consent for risky interventions	370	93.4	78	71.6
To decide whether to be treated in case of imminent death	130	32.8	N/A	
To have the best decision made in case of incapacity decision-making capacity	227	57.3	N/A	
To confidentiality of medical data				
With patient’s express consent	278	70.2	71	65.1
Without patient’s prior consent	129	32.6	51	46.8
To receive full access to their medical records	203	51.3	45	41.3

### Predictors of non-compliance with patient’s rights

The score for legal compliance with patients’ healthcare rights was computed to compare the physicians’ practices with their baseline characteristics. The mean score for attending physicians was 6.69 ± 1.99 out of 12, while general practitioners scored 5.39 ± 1.60 out of 9.

The overall behavior of the attending physicians was not significantly influenced by factors such as age, years in practice, work sector, or location. However, there were differences among attending physicians based on their specialty, with Anesth. Intensive Care physicians being less compliant compared to non-surgical physicians (Table 3). General practitioners’ behavior was similar across age (*p* = .136), years in practice (*p* = .667), and geographic area (*p* = .729).

**Table 3:** Attending physicians’ legally compliant behavior according to baseline characteristics

Variables	N	MS	SD
Specialty*			
Anesth. Intensive Care	24	5.63	1.93
Surgical	96	6.70	2.22
Non – surgical	181	6.90	1.81
ObGyn	71	6.87	1.53
Emergency	24	5.54	2.84
Age			
< 30	27	5.70	1.86
30–39	129	6.65	1.88
40–49	102	6.84	2.14
50–59	74	6.88	2.09
> 60	31	6.61	1.63
N/A	33	6.79	2.00
Years in practice			
1–5	75	6.21	1.91
6–10	60	7.05	1.99
11–15	58	6.59	1.96
16–20	48	6.58	2.14
21–25	52	7.04	2.08
26–30	37	6.92	1.92
> 30	31	6.32	1.60
N/A	35	6.94	2.13
Work sector			
Public	188	6.89	1.93
Private	208	6.50	2.03
Location			
Dolj	305	6.57	1.87
Olt	91	7.08	2.30

*p < .005

### Paternalistic behavior

Regarding patient autonomy, results indicated that emergency physicians were less likely to seek written consent for these procedures compared to other specialists (*p* < .001, df = 1, V = 0.49, λ = 0.08). Specifically, only 45.8% of emergency physicians sought written consent for interventions that posed a risk, compared to over 90% of other specialists. The remaining emergency physicians only requested written consent when the risk was high. Similarly, only 33.3% of emergency physicians sought informed consent for collecting biological samples. Additionally, physicians working in the public sector were significantly more inclined to deny patients’ right to consent to treatment and declared to terminate the therapeutic relationship in this case (*p* = .023, df = 1, V = 0.12).

In terms of patient privacy, 50.4% of general practitioners and 62.4% of attending physicians acknowledged communicating treatment information to family members and companions When it came to sharing medical records with the patients, anesthesia and intensive care physicians and general practitioners were found to be less willing to communicate all information. In this case, most anesthesia and intensive care physicians (66.7%) and a significant part of the general practitioners (46.8%) permitted access to only diagnosis and treatment information.

### Allocation of scarce resources

**Fig. 1:**
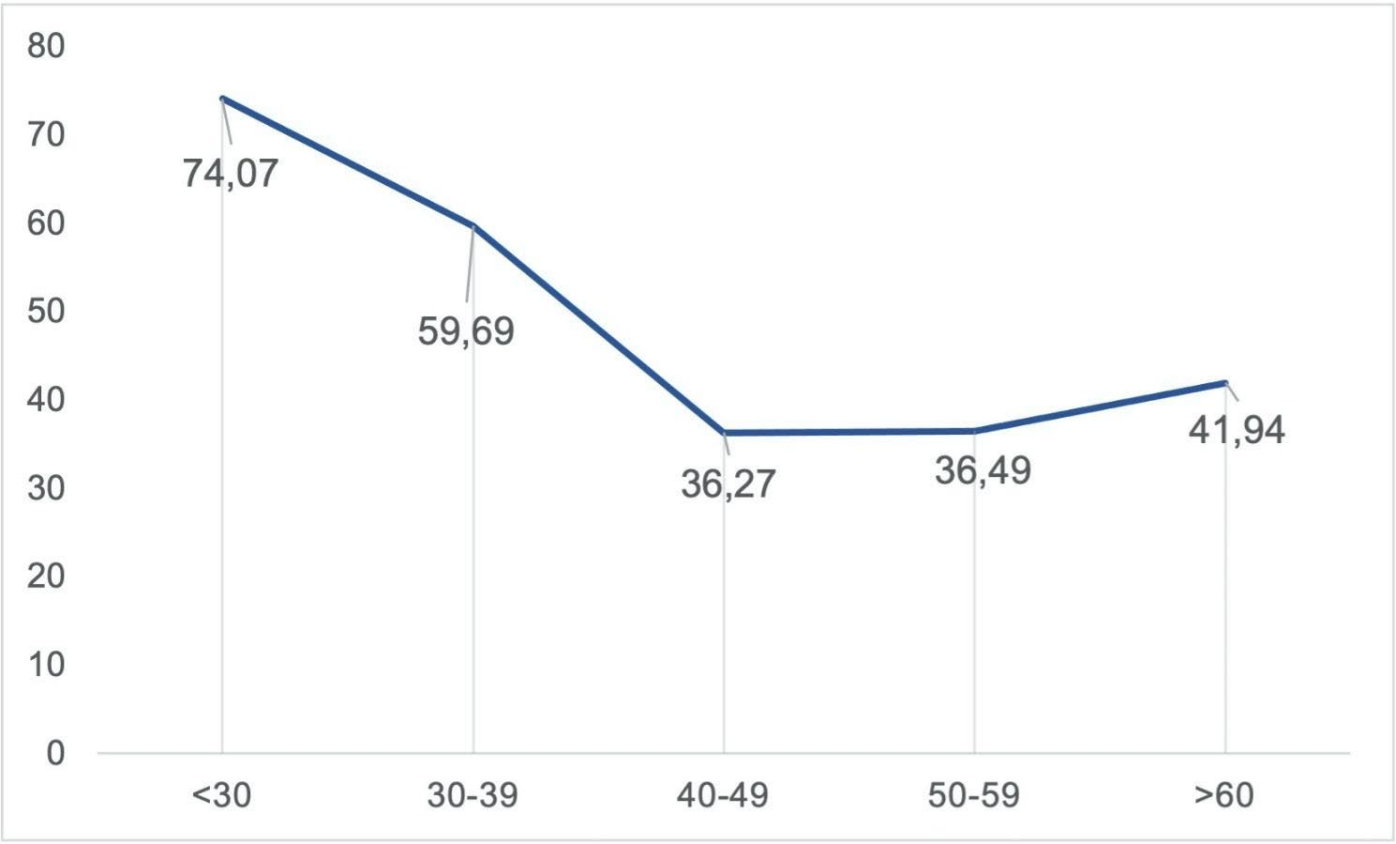
Comparison of Physicians’ Preference for Younger Patients Across Age Groups, Legend: x-axis: Age Categories; y-axis: Percentage of Physicians Prioritizing the Youngest Patient

Results showed that when asked to prioritize treatment for patients with similar medical conditions, 46.2% of attending physicians prioritized the younger patients. A Chi-Square Test for Independence revealed significant associations between prioritizing the youngest patient and attending physicians’ age (*p* < .001, df = 4, V = 0.17, λ = 0.22), years in practice (*p* < .001, df = 4, V = 0.18, λ = 0.22) and specialty (*p* = .031, df = 4, V = 0.14, λ = 0.08). The attending physicians that prioritized the younger patient had a median age of 38 and a median experience of 10 years. The attitudes of physicians under 39 years towards prioritizing treatment for younger patients differed significantly from those between 40 and 59 years (Fig. 1).

Significant differences were also noted between specialties, with 70.8% of Anesth. Intensive Care physicians favoring younger patients compared to 38.5% of surgeons. The results indicate that general practitioners had a relatively consistent attitude toward prioritizing patients, with only 28.4% considering age as a factor. No significant variations were observed.

The results of the human resources analysis show that over half of the attending physicians (n = 221) reported having to work outside the scope of practice due to staff shortages. Additionally, 93 attending physicians reported treating patients outside their expertise but did not attribute it to staff shortages. They instead reported that this was a routine practice that occurred after consulting with colleagues before providing treatment.

The study results show that 27.3% of attending physicians reported working outside their expertise on more than ten occasions during the pandemic. There was a small size effect correlation between physicians who admitted to working outside their expertise more than ten times and those who reported committing legal breaches (*p* = .008, df = 1, V = 0.16, λ = 0.04). The directional measures indicate that knowing a physician’s response to legal breaches can increase the chances of predicting if they would work outside their scope of practice by 4%. But admitting to working outside the scope of practice would give no prediction to admitting to legal breaches. Additionally, no correlation was found between practicing outside of one’s expertise and the fear of malpractice or patient claims among physicians.

### Malpractice risks

9.8% of attending physicians admitted to being accused of malpractice. There were significant variations between specialties (*p* <. 001, df = 1, V = 0.23, λ = 0.05), age (*p* = .003, df = 1, V = 0.21, λ = 0.03), and years in practice (*p* < .001, df = 1, V = 0.25, λ = 0.02). Anesthesia Intensive Care and surgical physicians, physicians practicing for more than 30 years, and physicians over 60 years old were the most frequently accused of malpractice. On the other hand, non-surgical and emergency physicians and those between 30 and 39 years old with one to five years of professional experience were the least likely to be accused of malpractice. No correlations were found between physicians’ behavior, admitting to law breaches, and malpractice claims.

The study found that the fear of malpractice among attending physicians was linked to their specialty, not to other factors such as behavior, age, years of practice, working outside their expertise, previous malpractice claims, or admitting to legal breaches. Non-surgical physicians were the least affected by fear of malpractice claims, while emergency physicians were the most affected. Additionally, the general physicians working in urban areas were more afraid of malpractice than those working in rural areas (*p* = .029, df = 1, V = 0.23). Both attending and general practitioners cited work pressure as the main effect of being afraid of malpractice claims (Table 4).

**Table 4:** Physicians’ response to self-assessment questions

Malpractice risks	Attending Physicians		General Practitioners	
	No	%	No	%
Malpractice claims				
Yes	39	9.8	4	3.7
No	354	89.4	104	95.4
Malpractice fear				
Yes	302	76.3	97	89
No	77	19.4	10	9.2
Malpractice Fear Effect				
No effect	184	46.5	44	40.4
Work pressure	174	43.9	57	52.3
Patient relationship deterioration	38	9.6	9	8.3
Refuse high-risk interventions	27	6.8	N/A	

## Discussion

During the Covid pandemic, Romanian physicians showed limited adherence to basic patients’ rights while facing significant pressure due to the fear of malpractice claims. Patients’ right to receive medical information was generally respected, but confidentiality was moderately observed since sharing treatment information with family members and acquaintances was common practice. A similar study was conducted in Iran during the pandemic. This study produced similar findings, indicating that patients had high levels of access to information, but their privacy rights were not adequately respected [41]. Furthermore, physicians’ specialties significantly impacted their attitudes toward patients. The observed significant disparities in legal compliance between Anaesth. Intensive Care and non-surgical physicians could be attributed to the unusual circumstances posed by the pandemic. Specifically, Anaesth. Intensive Care physicians faced a considerable number of critically ill patients, high levels of stress, and burnout that could result in suboptimal patient care practices [42, 43]. In contrast, non-surgical physicians were comparatively less engaged in the pandemic due to limitations on hospital admissions for patients with chronic diseases. Surgeons and Anaesth. Intensive Care physicians reported the highest incidence of malpractice accusations, as the likelihood of patients’ accusations increases when adverse events occur [44].

Romanian physicians prioritized beneficence over autonomy in the therapeutic relationship, leading to a paternalistic approach during the pandemic [45]. This was demonstrated by their reluctance to accept patient decisions to refuse treatment and, in situations of imminent death, seeking consent from relatives or other physicians. In non-emergency situations, they choose to terminate the relationship with the patients. These findings align with prior research, which showed that even outside of pandemic conditions, the principle of life protection was ranked above autonomy and freedom [46].

The results indicated a significant improvement in physicians’ legal compliance, while malpractice fear and malpractice accusations decreased over the past ten years [20, 29]. The most notable progress was observed in the area of patient explicit consent, with a 30% increase in attending physicians who nowadays require written consent for biological samples and risky procedures compared to the previous study. This increase in legal compliance could likely be attributed to mandatory hospital procedures through hospital accreditations in the absence of any improvement in the university educational curriculum. Poland, another ex-communist country, which has also been identified as having weak enforcement of patients’ rights, shows better compliance in comparison [47]. Studies indicated that only a third of Polish physicians violated patients’ privacy rights, and less than 3% refused patients access to their medical records outside of Covid-19 situations, while almost half of the Romanian physicians did so [48]. There may be various factors that explain the discrepancies in physician compliance between the two countries, including factors beyond the pandemic. For example, in Poland, the existence of an ombudsperson to advocate for patients’ interests and the high level of awareness among Polish patients regarding their rights may have influenced physician behavior and forced them to respect patients’ rights [47, 49].

Resource scarcity was a major concern during the pandemic. Even well-developed countries struggled to offer proper care to all patients due to a lack of essential medical materials and personnel. The issue of resource allocation was particularly complex when it came to prioritizing patients for treatment. While some suggested using criteria such as age, medical condition, survival chances, and costs, others proposed principles such as the number of lives and years to live, favoring patients with a greater probability of survival, the greatest medical benefit for the greatest number of patients and randomization [50–54]. However, it was generally agreed that age, disability, or medical condition should not be the only criterion employed, and physicians should avoid working outside their expertise despite resource scarcity [51]. Romanian in-force legislation prohibited discrimination on age, nationality, sex, income, or any other criteria and failed to adjust to pandemic conditions and offer solutions in case of resource depletion. However, results indicate that the shortage of material resources caused older patients’ discrimination when competing for resources, determining healthcare access and outcomes disparities. The staff shortage forced more than half of the physicians to work outside their scope of practice, causing pressure and affecting patient care quality.

During the pandemic, Romanian healthcare professionals saw justice as the principle most frequently violated [45]. Compared to the 2007–2008 and 2009–2013 surveys, discrimination on age criteria increased [20, 29]. For instance, when asked how they chose between two patients in similar conditions, 68% of physicians would not differentiate on age criteria in previous studies, as opposed to 49% of our respondents during the pandemic. The increased in-group favoritism and ageism could only be attributed to the pandemic conditions and was recorded in many other countries such as Britain, Sweden, Germany, France, Italy, and the Netherlands, although to varying degrees [53, 55, 56]. Notably, even physicians who were uncomfortable employing the age criteria would apply it when old age was a determinant for a poor outcome [27]. Additionally, some studies obtained similar results indicating that the use of age criteria decreased with respondents’ age, and younger patients were favored by young and elderly respondents [55, 57].

### Strengths and limitations

The study reported a significant effect size of the findings, thus indicating that the findings are not due to change and necessitate further investigations. Research points to the necessity of patient triage regulations in the event of resource scarcity and legal rules referring to physicians’ competence in case of personnel shortage. Moreover, the results indicate that compliance with patients’ rights needs to be increased, and exploration of the causes of low compliance is further needed.

Although the study is one of the few that addressed physicians’ routine practice during the pandemic and focused only on physicians who provided care to patients during the pandemic, it has some limitations due to methodological and practical constraints. For instance, it covered only some patient rights-related aspects and was concentrated on one region in Romania. Although we assume a behavioral change in the case of Romanian physicians due to the pandemic context, we could not explain how much of the Romanian physicians’ practices could be attributed to the pandemic or standard hospital practices. Further research is needed to assess physicians’ attitudes toward patients’ rights in normal circumstances and possible causes of non-compliance. Furthermore, due to a lack of data from the College of Physicians, we could not calculate the sample population according to demographic variables.

## Conclusion

These findings may aid future healthcare decision-makers and hospital administrations in implementing additional measures to ensure a better compliance rate. The high level of malpractice fear affected a large part of physicians during the pandemic, while the patients’ rights were suboptimal. Due to the profound impact on physicians and patients, it is most important for the authorities involved to design preventive policies and interventions that improve physicians’ compliance and deal with the increased pressure and defensive medicine effects caused by malpractice fear. Additionally, a lack of political decisions in the resource depletion scenarios determined discrimination based on age criteria and forced physicians to perform outside their expertise. To address the identified areas of low legal compliance among physicians and ensure better respect for patients’ rights, the educational curriculum should be improved by introducing mandatory medical law and ethics classes. By doing so, the medical profession can improve patients’ outcomes and increase their confidence in the healthcare system.

## Electronic supplementary material

Below is the link to the electronic supplementary material.

Supplementary Material 1: Attending Physicians’ Questionnaire

Supplementary Material 2: Attending Physicians’ Questionnaire

## Data Availability

Data are available at the corresponding author upon reasonable request.

## Abbreviations

Anaesth. Intensive Care: Anaesthesia and Intensive Care
ObGyn: Obstetrics–Gynecology
