# Supplementary material for: Patients’ rights in physicians’ practice during Covid-19 pandemic: a cross-sectional study in Romania
Source: BMC Med Ethics. 2023 Jul 26;24:54. doi: 10.1186/s12910-023-00935-8 (PMC10373321; doi:10.1186/s12910-023-00935-8)
Supplement: Supplementary file 2 — Supplementary Material 2: Attending Physicians’ Questionnaire [file 12910_2023_935_MOESM2_ESM.pdf]

Questionnaire / Correct answers for general physicians
(Translated into English)

The research aims to identify the main vulnerabilities of medical practice in the face of potential malpractice accusations, reduce professional risks, and avoid medical malpractice situations. Please answer the questions accordingly to your practice.
The questionnaire consists of 14 questions. You can either check one, more, or none of the answers. To achieve the objective, it is necessary for your response to reflect the way you currently proceed in your practice.
Your answers are completely anonymous, and we do not collect, in any way, personal data.

I agree
I declare to be a physician with the right of practice and that I have not previously filed this questionnaire: a) Yes, b) No.

Section 1: Information on the research participants:

1. Years in practice: ____
2. Age: _____
3. Location: ☐urban, ☐rural

Question no. 1: Is the information about the health state of a patient with decision-making capacity communicated to a third party?" Proposed answer options: a) Yes, only to close relatives. b) No. c) Yes, with the express acceptance of the patient.
*The correct answer envisaged: "Yes, with the express acceptance of the patient" (c).*

Question no. 2: "Is the information about the treatment followed by a patient communicated to a third party?" Proposed answer options: a) Yes if they accompany the patient to hospital admission, b) Yes, to the patient's family members. c) No.
*The correct answer envisaged: "No" (c).*

Question no. 3: „Between two patients with similar medical conditions, which one do you prioritize?". Proposed answer options: a) The youngest patient. b) The Romanian patient. c) The patient with a higher economic status.
*The correct answer envisaged: none*

Question no. 4: “You are about to perform a potentially risky procedure on the patient. Do you explain the risks to the patient?” Proposed answer options: a) Yes, always, in detail. b) No, to protect the patient. c) No, the decision made exclusively belongs to the doctor.
*The correct answer envisaged: “Yes, always, in detail” (a).*

Question no. 5: “Have you obtained the patient's written consent for risky procedures?” Proposed answer options: a) No, this decision belongs to the doctor.
b) Yes, always. c) Only when it involves major risks.
*The correct answer envisaged: “Yes, always” (b).*

Question no. 6: „How do you proceed when a patient requires medical intervention beyond your competency?”. Proposed answer options: a) I do not intervene no matter the patient's status. b) I offer medical support after consulting with a competent physician. c) I offer treatment in case of an emergency.
*The correct answer envisaged: I offer treatment in case of an emergency. (c)*

Question no. 7: “Has the patient access to his/her medical information about the investigations performed, diagnostic, and recommended treatment?” Proposed answer options: a) Not necessary; when the patient is released from the hospital, he/she is informed about the treatment scheme and the necessity of returning for medical checks. b) Yes, always, completely. c) Only information about the diagnostic and treatment methods is made available to the patient.
*The correct answer envisaged: “Yes, always, completely” (b).*

Question no. 8: „ You may terminate the therapeutical relationship with one of your patients:” Proposed answer options: a) After accepting him/her as a patient, the relationship may be terminated only when the patient is cured. b) when the patient refuses recommended investigations or treatment. c) when the patient manifests a hostile or irreverent attitude.
*The correct answer envisaged: „When the patient manifests a hostile or irreverent attitude.” (c)*

Question no. 9 “Biological samples are collected and tested:” Proposed answer options: a) Based on the doctor’s decision, exclusively. b) After acquiring the patient’s consent. c) After consulting the management of the healthcare unit, based on medical and financial criteria. *The correct answer envisaged: “After acquiring the patient’s consent” (b)*

Question no.10: “The patient cannot express his/her consent, and his/her health condition requires emergency procedures. The patient’s consent file is substituted with:”
Proposed answer options: a) The patient’s consent is not necessary for emergency situations. b) A written report produced by the doctor who provides the medical care, attached to the patient’s consultation sheet. c) The decision is made by the board of the medical unit.
*The correct answer envisaged: “A written report, produced by the doctor who provides the medical care, attached to the patient’s consultation sheet” (b)*

Self-assessment:

Question no. 11: „Have you performed medical acts that could be considered a law violation in the past three years?” Proposed answer options: a) yes, b) no.

Question no. 12: „Has a patient filed a complaint against you alleging malpractice?”. Proposed answer options: a) Yes. b) No

Question no. 13: „Do you view patients’ accusations of medical malpractice as a serious and present threat?”. Proposed answer options: a) Yes. b) No

Question no. 14: „Has a fear of malpractice accusations affected your activity in any way?” Proposed answer options: a) No. b) Yes, I have noticed a deterioration in the relationship with patients. c) Yes, it has led to an increase in pressure during interventions.
